# Rev. J. Davis Gray

**Published:** 1887-03

**Authors:** 


We are pained to hear of the death of Dr. J. A. Gray's brother, Rev. J. Davis Gray, who died at Hawthorne, Florida, on 21st February, ult.

---

During the session of the Texas State Medical Association, this JOURNAL will issue a daily edition, for gratuituous distribution to members. The April number will contain a handsome cut of the late Dr. Burt.

